# Identifying co-occurring neighbourhood environmental patterns and their association with health behaviours in a Dutch urban population at high cardiometabolic risk

**DOI:** 10.1186/s12889-026-28175-6

**Published:** 2026-07-07

**Authors:** Anne K. Smit, Rimke C. Vos, Laura A. van der Velde, Marcel R. Haas, Mattijs E. Numans, Mariëlle A. Beenackers, Tobias N. Bonten, Jessica C. Kiefte-de Jong

**Affiliations:** 1 Department of Public Health and Primary Care (V-0-P), Health Campus The Hague, Leiden University Medical Center, PO Box 9600, The Hague, 2300RC The Netherlands; 2 Department of Public Health, Erasmus MC, University Medical Center, Rotterdam, The Netherlands

**Keywords:** Built environment, Food environment, Green space, Walkability, Cardiovascular disease

## Abstract

**Background:**

Pattern-based approaches to characterising neighbourhood environments have been applied across cities and regions, but it is less clear whether they can identify independent environmental effects within a single dense urban area, the scale at which most local public-health interventions are designed. Furthermore, research has primarily focused on general populations, leaving a gap in understanding how environmental factors relate to health behaviours in high-risk groups. This study aimed to (1) describe how a broad set of food-environment, green-space and walkability indicators co-occur in a highly urbanised Dutch city and how the resulting patterns relate to population density and neighbourhood socioeconomic position; and (2) explore whether these patterns are associated with weight status, diet quality and physical activity in primary-care patients at high cardiovascular risk.

**Methods:**

This cross-sectional study used baseline data from the Healthy Heart study in The Hague, Netherlands (*N* = 475 participants from 73 postal codes). Twenty-three indicators across food environment, green space and walkability domains were assessed at two spatial scales. Correlation analyses examined intercorrelations among indicators and contextual characteristics. Neighbourhood patterns were identified using principal component analysis (PCA). Associations of component scores with weight status, diet quality and physical activity were examined using (multinomial) logistic and linear regression, adjusted for individual and neighbourhood-level covariates.

**Results:**

Four components explained 87% of the variance in the environmental indicators. The dominant neighbourhood pattern, characterised by high food-retail density, high walkability and low vegetation cover, correlated very strongly with population density and this coupling persisted within the most highly urbanised stratum. The “Relative food environment advantage” pattern showed the most consistent association across models with lower odds of being overweight, but also counterintuitively with lower fruit and vegetable diet quality. There was some indication of effect modification by neighbourhood socioeconomic position for this pattern.

**Conclusions:**

Within a single dense urban area, neighbourhood characteristics cluster into structured patterns in urban settings. Food environment measures remained coupled to population density even within high-density areas, fundamentally challenging independent effect estimation. Modest, paradoxical associations suggest limited environmental influence on lifestyle behaviours and weight in medically supervised high-risk populations.

**Supplementary Information:**

The online version contains supplementary material available at https://doi.org/10.1186/s12889-026-28175-6.

## Background

Cardiovascular disease (CVD) remains a leading cause of morbidity and mortality globally, with a substantial proportion of the population at high risk for developing CVD [1]. The physical neighbourhood environment has been linked to cardiovascular outcomes. For instance, the density of fast-food outlets has been linked to poor diet quality, higher BMI, and less physical activity [2–4], all of which contribute to CVD risk. Similarly, exposure to green spaces has been associated with better cardiovascular outcomes, including reduced incidence of CVD events, lower BMI, and higher levels of physical activity [2, 5]. However, as pointed out in several reviews, these associations often remain weak and are complicated by methodological issues [2, 5–7]. 

A first methodological challenge is that many studies examine the impact of single environmental exposures in isolation, such as the density of fast-food outlets or proximity to green spaces, neglecting the complex interplay between neighbourhood characteristics. The view that neighbourhoods should be understood as configurations of interrelated features rather than collections of independent exposures has been articulated in broader conceptual frameworks [8, 9] and has led to an increasing body of work developing multivariable operationalisations of the neighbourhood environment, including walkability indices [10, 11], composite obesogenic-environment indices [12], and data-driven approaches using methods like principal component analysis (PCA), latent class analysis (LCA) or cluster analysis [13–15]. Many of these studies have been conducted on large national or multi-city datasets, which may derive much of their explanatory power from variation in population density, socio-economic conditions and urban form between settings, variation that is much narrower within a single city. Whether pattern-based decomposition applied within a single dense urban area, the scale at which local public-health policy is designed and delivered, produces environmental dimensions that are independent of this underlying population density and socio-economic conditions is less well examined.

A second challenge is the lack of consensus on how to define and measure these environmental exposures. For the food environment, studies have used density measures (e.g., number of outlets in a buffer), accessibility measures (e.g., network distance to nearest retailer), and relative measures such as the Modified Retail Food Environment Index (MRFEI), which compares the healthy to unhealthy availability [3, 7]. Similarly, for green space exposure, measures include density (e.g., percentage of green space in buffer), accessibility (e.g., distance to nearest park), and quality indicators such as the Normalised Difference Vegetation Index (NDVI) [16, 17]. Studies comparing different operationalisations of the same environmental constructs often find divergent results, suggesting these measures may capture distinct environmental dimensions rather than interchangeable alternatives [18–20]. 

Beyond these measurement challenges, most existing research has focused on the general population from population-based cohort studies with relatively healthy individuals [7, 16]. This leaves a significant gap in our understanding of the interplay between environmental factors and lifestyle behaviours in high-risk groups, such as those already receiving care. From a public health perspective, population-wide interventions are logical, as they yield larger absolute health gains. However, a substantial and growing portion of the population is already at high risk for developing CVD, and these individuals are typically engaged in primary-care-based prevention programmes offering lifestyle counselling and medical follow-up that may modify the influence of neighbourhood exposures. For these individuals, understanding which factors are most salient could inform targeted interventions. Studies in high-risk populations are therefore crucial for determining which factors warrant prioritisation, particularly those linked to adverse health outcomes in both the general and high-risk population [21, 22]. 

This study therefore has two aims: (1) to describe, within a single highly urbanised area, how a broad set of food environment, green space and walkability indicators (covering multiple operationalisations at two spatial scales) co-occur, and how the resulting neighbourhood patterns relate to population density and neighbourhood socioeconomic position; and (2) to examine whether these patterns are associated with weight status, diet quality and physical activity in primary care patients at high cardiovascular risk.

## Methods

### Study design and setting

This study was conducted as a secondary analysis using baseline data from the Healthy Heart study, a non-randomised cluster stepped-wedge trial investigating the effect of a lifestyle program on patients in the primary cardiovascular prevention care protocol in general practice in the Hague [23]. The Hague is the third largest city of the Netherlands, with approximately 540.000 residents and characterised by dense urban development.

The Healthy Heart study was approved by the Ethics Committee of the Leiden University Medical Centre (P17.079). All participants provided written informed consent.

### Study area and spatial unit definition

Environmental characterisation was conducted for 73 four-digit postal codes where participants resided: 48 in The Hague (65.8%) and 25 (34.2%) in directly adjacent municipalities, reflecting typical catchment areas of general practices in the Hague. Because postal codes vary in size, environmental indicators were first calculated at the address level using residential address locations from the national Registration of Addresses and Buildings (BAG) [24], and then averaged at the postal code level. We included indicators at two spatial scales: within 300 m (immediate neighbourhood) and within 300–1000 m (extended neighbourhood, calculated as a donut-shaped buffer). The 300–1000 m buffer was constructed as a donut to separate the immediate (0–300 m) from the extended (300–1000 m) neighbourhood, allowing the PCA to empirically test whether these spatial scales capture similar or distinct environmental dimensions, rather than nesting one within the other.

### Environmental exposure measures

Geospatial analyses were performed using Qgis version 3.18.3 to calculate the 23 indicators presented in Table 1. For an extensive description of the geospatial analyses, see Additional File 1.

**Table 1:** Included environmental exposure variables

Category	Variable	Calculation	Variable Name
Food environment	Density of core food retailers in buffers	Number of core retailers in buffer	Core_300m, Core_donut
	Density of non-core food retailers in buffers	Number of non-core retailers in buffer	Noncore_300m, Noncore_donut
	Modified retail food environment index (MRFEI) in buffers* [25]	(Number of core retailers + 1) / ((Number of core retailers + 1) + (Number of non-core retailers + 1)) × 100	MRFEI_300m, MRFEI_donut
	Food environment health index (FEHI) in buffers [26]	Sum of FEHI-scores of food retailers in buffer	FEHI_300m, FEHI_donut
	Spatial access to core-retailers in buffers	Accessibility score to core retailers	Spac_core_300m, Spac_core_donut
	Spatial access to non-core retailers in buffers	Accessibility score to non-core retailers	Spac_noncore_300m, Spac_noncore_donut
	Ratio of spatial access scores in buffers* [27]	(Spatial access to core retailers + 1) / ((Spatial access to core + 1) + (Spatial access to non-core retailers + 1))	Rel_spac_300m, Rel_spac_donut
Green spaces	Density of accessible green spaces in buffers	Percentage accessible green space in buffer	Perc_access_green_300m, Perc_access_green_donut
	Density of other green spaces in buffers	Percentage other green space in buffer	Perc_other_green_300m, Perc_other_green_donut
	Network distance to nearest entrance of accessible green space	Shortest walking distance (in meters) to nearest entrance of accessible green space	Nearest_park_network
	Mean Normalized Difference Vegetation Index (NDVI) in buffers	Average NDVI value in buffer area	NDVI_300m, NDVI_donut
Walkability	Walkability score in 4 digit postal code [11]	Composite walkability index score	Walkability_250m, Walkability_1000m

*To avoid division by zero in areas with no food retailers, a score of one was added to the number of core and non-core retailers

### Food-environment

Food-environment data, published in 2019, was obtained from Locatus, a company that collects information on business locations through regular field audits [28]. Following previous example we calculated: density of core and non-core food retailers (counts of retailers per buffer) [26], total Food Environment Healthiness Index (FEHI) score (sum of healthiness score for all food retailers per buffer) [26], spatial accessibility to core and non-core retailers (sum of inverse Euclidean distances to all respective retailers within buffer), the Modified Retail Food Environment Index (MRFEI, percentage of core food retailers out of all retailers in buffer) and relative spatial accessibility (ratio of spatial access to core food retailers out of spatial access to all food retailers) [25, 27]. 

### Green spaces

Four indicators were assessed using Dutch Land Use data (2017) [29] and Landsat 8 satellite imagery [30]: percentage of accessible green space (e.g. parks, forests) in buffer, percentage of other green space (e.g. sports fields, agricultural land), network distance to nearest accessible green space entrance (shortest walking distance via road network), and mean Normalised Difference Vegetation Index (NDVI) within buffer.

### Walkability

As an additional environmental factor, the Dutch walkability index developed by Lam et al. [11]. obtained through the Geoscience and Health Cohort Consortium (GECCO) was used [31, 32]. This composite score, which can be used to assess the degree to which an area is conducive to walking as a mode of transportation, includes population density, density of retail and service destinations, land-use mix, street connectivity, green space and sidewalk presence. The walkability index was calculated by averaging the z-scores of these components, and then scaling the result to range from 0 to 100. The index was previously positively associated with walking behaviour in Dutch adults [11]. Data, for the year 2017, was pre-processed on address-level by GECCO and aggregated on average score in a 250 m and 1000 m buffer.

All 23 indicators were included in the PCA, including multiple operationalisations of the same constructs (e.g., density, accessibility, and relative measures of the food environment) and two spatial scales (300 m and 300–1000 m buffer). PCA was chosen because (i) it allows empirical testing of whether different operationalisations of the same construct (e.g., MRFEI vs. FEHI for food environment) capture similar dimensions; (ii) it reduces the multicollinearity among 23 indicators; and (iii) it yields interpretable composite scores at the postal code level that can be linked to participant data.

We distinguish in our analyses between environmental indicators (the 23 measures of food environment, green space and walkability) and contextual neighbourhood characteristics: population density and neighbourhood socioeconomic position (see also under correlation analysis and potential confounders). The latter are structural upstream features that shape both the distribution of environmental indicators and individual outcomes. They are therefore handled separately: descriptively (correlation analyses) and as adjustment variables in regression models.

For further details on the included indicators, see Additional File 1.

### Correlation analysis

To explore relationships between environmental indicators and contextual neighbourhood characteristics, a correlation analysis was conducted. Pairwise Spearman correlations were calculated between all environmental indicators and several contextual neighbourhood characteristics: urbanicity class (five levels, defined by Statistics Netherlands (CBS) [33]: very strongly urban (> 2500 addresses per km²), strongly urban (between 1500 and 2500 addresses per km²), moderately urban (between 1000 and 1500 addresses per km²), slightly urban (between 500 and 1000 addresses per km²), non-urban/rural (< 500 addresses per km²)), population density (continuous, using addresses per km² as a proxy; CBS 2017 [33]), and neighbourhood socioeconomic position (SES-WOA). The SES-WOA score is a composite indicator developed by Statistics Netherlands based on a combination of data regarding financial prosperity, educational level and recent employment history of the households, available per 4-digit postal code. Scores range between − 1 and 1, with higher values indicating higher neighbourhood SEP [34]. 

### Principal component analysis

Prior to PCA, variables were Box-Cox transformed to approximate normality and z-standardised (see Additional File 1 for details). To address multicollinearity issues and identify clusters of co-occurring neighbourhood features, a PCA with varimax rotation at the postal code level (*n* = 73) was performed, using the 23 standardized environmental indicators. To check sampling adequacy, Kaiser-Meyer-Olkin measure and Bartlett’s test of sphericity were used. The number of extracted components was based on eigenvalues greater than 1 and the scree plot. Varimax rotation was applied and rotated component scores were assigned to participants. For clarity, throughout the remainder of this article, the varimax rotated principal component scores will be referred to as the principal components (PC).

### Interpretation of PCA components

To explore how the environmental indicators relate to broader contextual neighbourhood factors, Spearman correlations were calculated between each continuous PC score (PC1–PC4) and two contextual neighbourhood variables that were not included in the PCA: Population density, and neighbourhood socioeconomic position (SES-WOA).

### Exploratory health associations

Having characterised environmental patterns in the neighbourhoods, we conducted exploratory analyses to examine whether these patterns were associated with health behaviours in a sample of residents at high cardiovascular risk.

#### Study population

Patients (> 18 years) at high risk for cardiovascular disease from 56 general practices in the Hague, Netherlands were invited to participate in the Healthy Heart study during a primary care consultation with their practice nurse or general practitioner (GP). For the current analysis, we included only baseline data (collected between June 2017 and April 2019) of patients included during the control period (before intervention implementation) who had a valid postal code, to avoid potential bias due to intervention participation. During the control period patients received care as usual.

Identification of high-risk patients is carried out as part of usual care in GP practices, without any involvement from the researchers. High-risk patients were defined as having a 10-year cardiovascular risk of ≥ 10% according to Dutch guidelines at the moment of inclusion (2017–2019) [23, 35]. As part of standard care, these patients have one to four individual consultations annually with a trained practice nurse. During these sessions, they receive lifestyle counselling, undergo lab work, and have physical examinations, all in line with Dutch Primary Care standards.

Patients with an ICPC coded diagnosis of diabetes mellitus or cardiovascular disease were excluded from participation in the general CVD prevention program, since they were already enrolled in separate care programs. Patients with significant palliative comorbidities were also excluded from participation. Data on sociodemographics, lifestyle behaviour and BMI were collected using self-reported online questionnaires.

#### Health outcome variables

Three outcomes were assessed: (1) diet quality, measured using the Dutch Healthy Diet index (DHD-index, range 0–160) [36, 37] based on the DHD-FFQ; (2) physical activity, measured using the SQUASH questionnaire [38] as total minutes per week; and (3) weight status, based on self-reported BMI categorised as healthy weight, overweight, or obese using age-specific cut-offs from the Dutch Nutrition Centre [39] (see Additional File 1 for more details). Outliers >3SD were removed. Missing DHD scores (6.7%) were addressed through zero imputation of subcomponents when no more than two of 16 were missing [40]. No imputation was performed for BMI (< 1% missing) or physical activity (< 2.5% missing).

#### Potential confounders

The following potential individual- and neighbourhood-level confounders were used in the regression analyses to adjust for associations between principal components and health outcomes. Individual confounders included: age (years), sex (male/female), level of education (lower, middle, higher) and migration background (yes/no). Neighbourhood level confounders included: population density (using continuous addresses per km² as a proxy) and SEP of the postal code (SES-WOA).

#### Statistical analysis of health associations

Sample characteristics were provided using descriptive statistics, summarized as percentage for categorical variables and mean (standard deviation) or median (IQR) for continuous variables, depending on distribution of the variable.

Intraclass correlation coefficients for all outcomes were < 0.05 (weight status = 0.02; DHD ≈ 0.00; physical activity = 0.01), indicating minimal clustering within postal codes; standard regression was therefore used without multilevel modelling.

For interpretability, the PCs were divided into quartiles prior to performing the regression analyses, as a one-unit change in PC score is not inherently meaningful. This decision was further supported by the absence of clear linear relationships between the PCs and outcomes.

The association between PC quartile and the DHD-score and total minutes of physical activity per week (square root transformed due to heteroscedasticity of the untransformed variable) were analysed using univariate and multivariate linear regression. The association between the PCs and weight status was analysed using a multinomial logistic regression (as the proportional odds assumption for an ordinal logistic regression was violated), with healthy weight set as the reference category. Assumptions were checked before conducting the analyses. Although population density correlated strongly with PC1 (continuous: *r* = 0.86, VIF = 8.6), quartile coding of PC1 reduced multicollinearity (VIF = 4.5).

In the univariate models, the PC quartiles were included individually (a separate model for each PC, without any further adjustments). The first multivariate model (model 1) also incorporated individual and neighbourhood level confounders. In the final model (model 2), all PCs were included simultaneously and adjusted for the individual and neighbourhood level confounders. Due to sample size considerations, the PC of interest was included in the model as a categorical (dummy or factor) variable, with the lowest quartile used as the reference category. This approach was consistent with how the PC was handled in the univariate model and model 1. In contrast, the other PCs that were used for adjustment (in model 2) were added as ordinal quartile scores (1–4). To establish dose-response relationships, linear trends (P-trend) across the quartiles were examined using the median values of each quartile as continuous variable in the analyses.

#### Additional analyses

Additional analyses of dietary and physical activity subcomponents, as well as effect modification by neighbourhood socioeconomic position, are described in Additional File 2.

### Sensitivity analyses

To characterise the extent to which postal-code aggregation altered the correlation structure of the environmental indicators with population density, address-level Spearman correlations were computed using all residential addresses within the 73 PC4s (*n* = 297,053).

Statistical analyses were performed in Python version 3.8 (packages factor analyzer, scikit-learn and SciPy) and SPSS version 29.

## Results

### Sample characteristics

Characteristics of the 475 participants are presented in Table 2. Participants were on average 65.6 years of age and 55.4% were female. A minority had a migration background (14.7%), most were middle to higher educated (69.4%), most participants below the age of 65 were employed (72.6%) and almost all participants lived in a (very) strong urban area (94.5%). Furthermore, 53.9% were either overweight or obese, and only 37.9% met the physical activity guideline of 150 min of moderate to heavy physical activity per week.

**Table 2:** Characteristics of participants (*n* = 475)

Characteristics		
Age (yr), mean (SD)	65.6 (9.3)	
Sex, female, n (%)	263 (55.4%)	
Migration background, n (%)	70 (14.7%)	
Educational level, n (%)		
Low	131 (27.6%)	
Middle	146 (30.7%)	
High	184 (38.7%)	
Employed (< 65 year), n (%)	151 (72.6%)	
Weight (kg), mean (SD)		
Weight status, n (%)	81.0 (16.1)	
- Healthy weight	213 (44.8%)	
- Overweight	156 (32.8%)	
- Obese	100 (21.1%)	
BMI (kg/m2), mean (SD)	27.1 (4.1)	
Total DHD-score, mean (SD)	98.4 (17.1)	
Total minutes of physical activity per week, mean (SD)	2045.5 (1200.1)	
Meets physical activity guideline, n (%)	180 (37.9%)	
Address density, median (IQR)	2836 (1865, 4130)	
Neighbourhood SEP*, median (IQR)	0.09 (-0.13, 0.20)	

*Measure of socioeconomic status at the local level, based on a combination of data regarding financial prosperity, educational level and recent employment history of the households [34]

The median address density was 2836 addresses per km² (range: 362–8499), corresponding to urbanicity class very strongly urban. The median neighbourhood SES-WOA score was 0.09 (range: − 0.98 to 0.58).

### Postal-code-level correlation analyses

Strong intercorrelations were observed among variables within the same exposure domains (Additional File 2, Figure A1a). Densities of core and non-core food retailers were very highly correlated (ρ ≈ 0.8), indicating that neighbourhoods with many non-core outlets tend to also have more core food retailers. Walkability was also strongly related to food-retailer density, particularly within the 1000 m buffer for walkability (ρ ≈ 0.8).

Relative food environment measures were strongly intercorrelated but showed lower correlations with absolute food density (not significantly correlated with core retailer density, significant negative correlation (ρ ≈ -0.4) with non-core retailer density and significant positive correlation with inverse FEHI (ρ ≈ 0.3–0.4)).

In contrast, green-space density and quality measures (but not network distance to nearest accessible green space) showed consistent negative correlations with food-environment and walkability indicators, reflecting that greener areas generally coincide with fewer retail destinations. Network distance to nearest accessible green space showed lower and non-significant correlations with other green space measures.

**Fig. 1:**
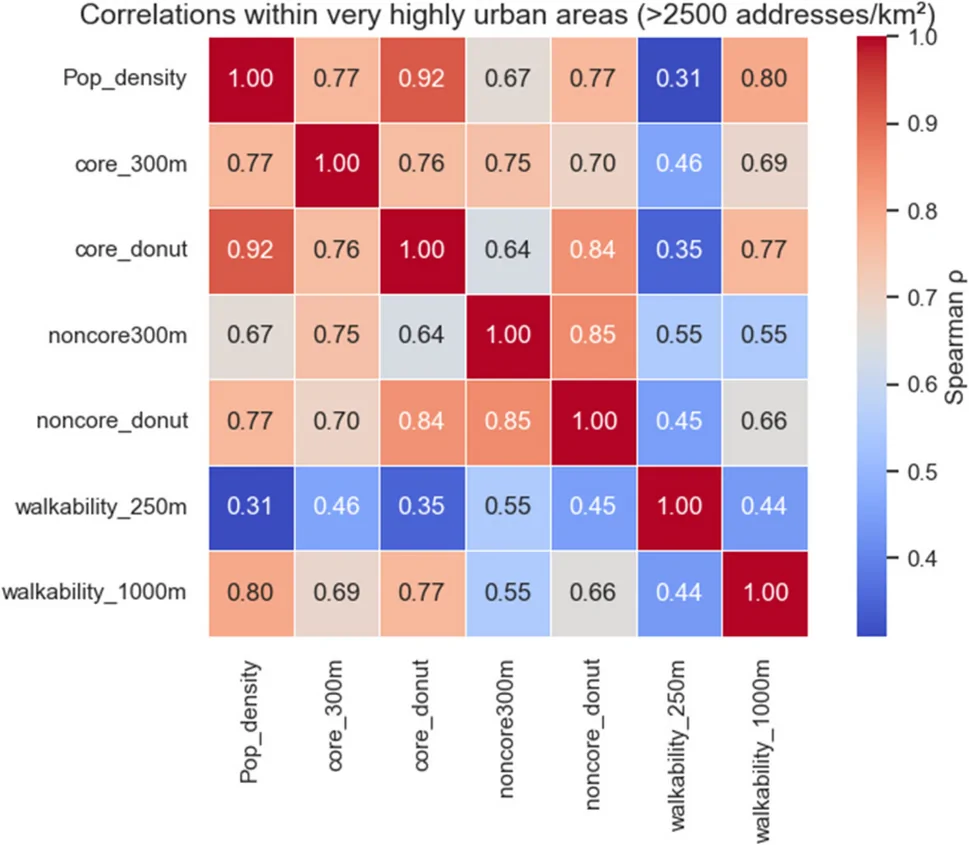
Correlation table within highly urban areas *For definition of the environmental indicators see Table 1. Pop_density (population density) defined as addresses per km^2^. SES: Measure of socioeconomic status at the local level, based on a combination of data regarding financial prosperity, educational level and recent employment history of the households [34]

When examining contextual characteristics, population density showed much stronger correlations with environmental indicators than the ordinal urbanicity classification. Population density correlated strongly with core retailer density (ρ ≈ 0.9), non-core density (ρ ≈ 0.8–0.9), and walkability (ρ ≈ 0.7–0.9), and inversely with NDVI and green-space indicators (between ρ = -0.3 and − 0.8). Even within the “very strongly urban” Statistics Netherlands category (> 2,500 addresses /km², *n* = 50 postal codes), substantial correlation remained: higher population density continued to be associated with higher retail density (between ρ = 0.7 and 0.9) (Fig. 1). Population density was not significantly correlated with the relative food environment measures.

Neighbourhood socioeconomic position showed somewhat weaker relationships with the environmental indicators: lower-SES areas tended to have higher retail density (between ρ = -0.4 and − 0.6) with lower FEHI-score and higher walkability (ρ = -0.6, ρ = -0.4) but less green space (although associations were low and only the association with accessible green space in donut buffer was significant (ρ = 0.2)).

Comparing the two spatial scales revealed that environmental characteristics in the immediate (300 m) and extended (300–1000 m, donut) buffers were generally highly correlated, indicating that neighbourhood features largely co-occur across scales in this urban area. The relative food environment measures showed lower correlations across scales (ρ ≈ 0.6).

### Address-level correlation (sensitivity) analyses

The overall correlation structure was broadly the same as at the postal-code level (Additional File 2, Figure A1b), but several aspects differed in informative ways.

The relative food environment measures continued to be uncorrelated with population density. Density confounding of absolute food environment indicators and walkability was also observed on address level but varied with buffer size. Donut indicators continued to be strongly correlated with population density (ρ ≈ 0.8), while 300 m buffer indicators showed substantially weaker density correlations (ρ ≈ 0.4–0.5). Cross-scale correlations between 300 m and donut versions of the same indicator were lower at the address level than at the postal-code level.

Restricting the address-level analyses (Additional File 2, Figure A1c) to the most highly urbanised areas (> 2500 addresses/km^2^) slightly reduced but did not eliminate density-confounding of absolute food environment variables (between ρ = 0.7 and 0.8 for donut variables and between ρ = 0.4 and 0.5 for 300 m variables).

### Principal component analysis

The Kaiser-Meyer-Olkin (KMO) measure confirmed sampling adequacy (KMO = 0.842), and Bartlett’s test of sphericity was significant (*p* < 0.001), indicating that the data were suitable for dimensionality reduction.

Four components had eigenvalues > 1 and were retained based on the scree plot, together explaining approximately 87% of the total variance (Additional File, Table A3).

### Component structure and interpretation

**Fig. 2:**
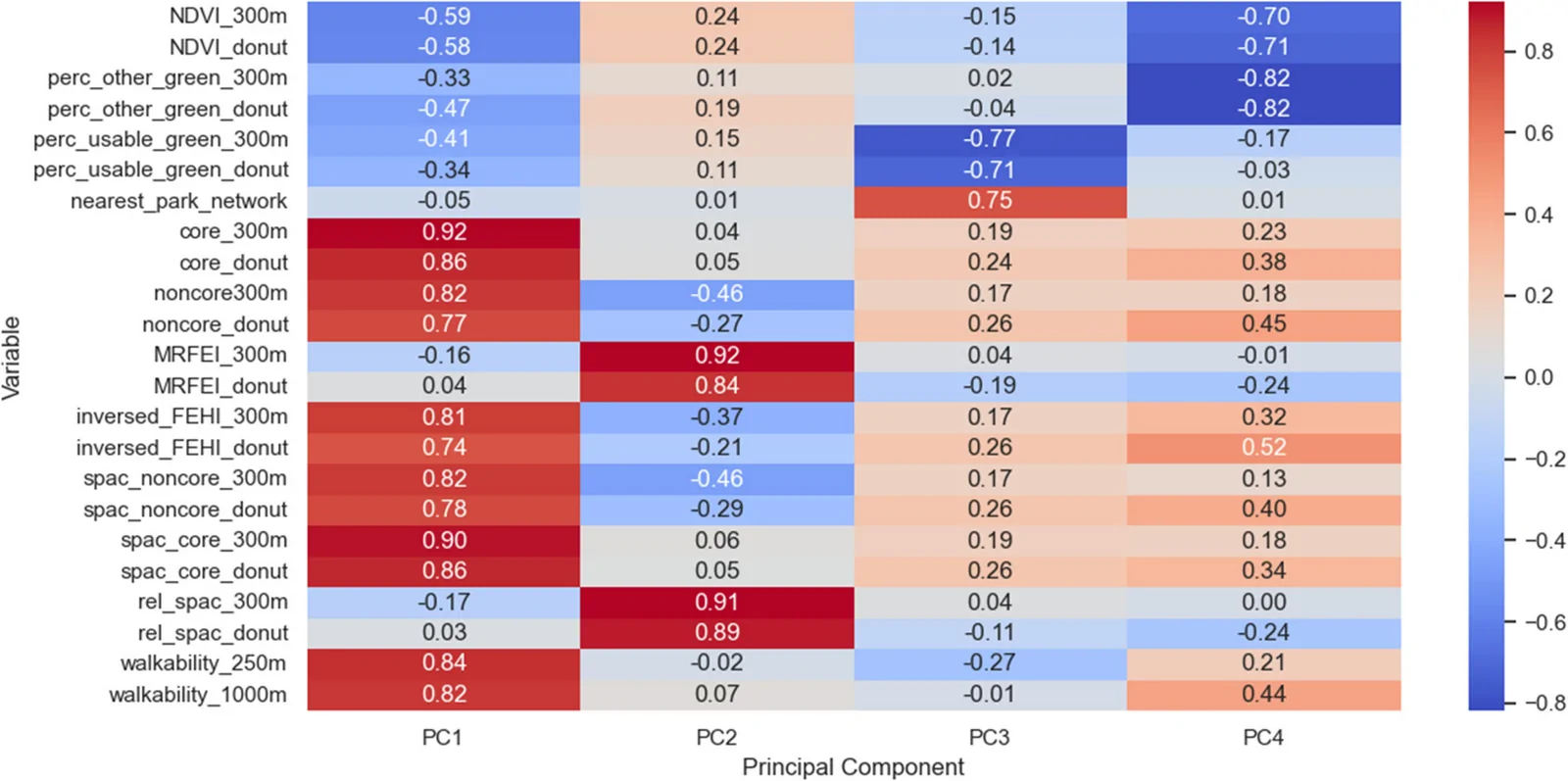
Variable loadings on the four varimax rotated principal components *NDVI = Normalised difference in Vegetation Index; perc_other_green = percentage of other green spaces, perc_access_green = percentage of accessible green spaces; nearest_park_network = shortest distance to entrance of nearest park; core = density of core retailers; noncore = density of noncore retailers; MRFEI = Modified Retail Food-Environment Index; inversed_FEHI = the inverse of Food Environment Healthiness Index; spac_noncore = spatial accessibility to noncore retailers; spac_core = spatial accessibility to core retailers; rel_spac = ratio of spatial access scores

The rotated component loadings (Fig. 2) revealed four distinct environmental patterns. For interpretability, component names describe the environmental characteristics of areas scoring in the highest quartile.

- PC1 (proportional variance explained: 41.8%) – “High food, low FEHI, high walkability”.
  This component had high positive loadings for both core and non-core food retailer densities, walkability, and inversed FEHI. It loaded negatively on NDVI at both scales and, more modestly, on percentages of accessible and other green space.
  It reflects highly urbanised environments characterized by dense food retailer presence (both healthy and unhealthy) and high walkability, but limited green space.
- PC2 (proportional variance explained: 18.0%) – “Relative food environment advantage”.
  This component loaded strongly on the relative measures (MRFEI, relative spatial access). It represents neighbourhoods where healthier food options dominate relative to unhealthy ones, largely independent of overall retail density (although non-core retailers and inversed FEHI at the 300 m buffer also loaded moderately on this component, indicating this dimension captures neighbourhoods where healthier options dominate not through greater absolute availability of healthy retailers but through lower density of unhealthy alternatives).
- PC3 (proportional variance explained: 10.1%) – “Low density and high distance to accessible green”.
  PC3 was defined by positive loadings for network distance to nearest accessible greenspace and negative loadings for accessible green space density, indicating low-density areas with poor accessibility to nearby green accessible space. This component therefore isolates a pattern of poor physical and network-based access to accessible green space, independent of overall vegetation or food environment characteristics.
- PC4 (proportional variance explained: 16.7%) – “Low-quality, agricultural/inaccessible green”.
  This component had high negative loadings for inaccessible (‘other’, mainly agricultural) green and NDVI, reflecting areas characterized by low vegetation quality and non-accessible agricultural green space. Additionally, it loaded moderately positively on several donut-scale food environment and walkability indicators, but only weakly on their 300 m counterparts. This component therefore reflects neighbourhoods with low vegetation cover and few non-accessible green areas, combined with moderately elevated unhealthy food retail and walkability in the extended (donut) buffer.

### Component correlations with contextual variables

To explore how these principal components related to broader neighbourhood context, Spearman correlations were computed between component scores and population density and neighbourhood socioeconomic position (See Additional File 2, Figure A2).

Population density showed a very strong positive correlation with PC1 (ρ = 0.9), while neighbourhood socioeconomic position was inversely correlated (ρ = − 0.6), indicating that the “High food, low FEHI, high walkability” pattern coincides with densely populated, lower-SEP areas.

Other components were only weakly associated with neighbourhood socioeconomic position or population density (|ρ| < 0.4).

These findings suggest that while the first component largely reflects a population density gradient, the remaining components capture neighbourhood environments that are largely independent of population density.

### Association with weight status

In both the univariate and the multivariate multinomial logistic regression analyses (Table 3), patients in higher quartiles of the “*Relative food environment advantage*” component showed statistically significant lower odds of being overweight (for Q3 (but not Q4) in multivariate model 2: OR = 0.45, 95% CI [0.22, 0.94], P-trend = 0.19). Patients in the third quartile of “*Relative food environment advantage*” also showed a lower risk of being obese in the univariate model and model 1, but this was no longer the case in model 2. These results suggest that neighbourhoods with relatively higher proportions of healthy food retailers were associated with a lower likelihood of being overweight, although no significant p for trend was found.

**Table 3:** Result of univariate and multivariate multinomial logistic regression analysis between varimax rotated principal components and weight status with healthy weight as the reference category

Weight status	Rotated Components Quartiles	Univariate analyses		Multivariate model 1		Multivariate model 2		*P for trend*
		*OR*	*95% CI*	*OR*	*95% CI*	*OR*	*95% CI*	
*Overweight*								
	High food, low FEHI, high walkability							0.86
	Q2	1.12	0.64, 1.97	1.25	0.66, 2.40	1.35	0.67, 2.72	
	Q3	1.15	0.66, 2.02	1.15	0.58, 2.29	1.21	0.51, 2.85	
	Q4	0.72	0.39, 1.33	1.04	0.41, 2.61	1.02	0.33, 3.16	
	Relative food environment advantage							0.19
	Q2	0.86	0.48, 1.53	0.79	0.41, 1.52	0.82	0.42, 1.59	
	Q3	0.50*	0.27, 0.93	0.48*	0.24, 0.94	0.45*	0.22, 0.94	
	Q4	0.72	0.39, 1.30	0.68	0.34, 1.37	0.66	0.32, 1.36	
	Low density and high distance to accessible green							0.93
	Q2	1.08	0.60, 1.91	0.93	0.49, 1.79	1.04	0.51, 2.13	
	Q3	1.10	0.62, 1.96	1.51	0.80, 2.84	1.59	0.83, 3.05	
	Q4	1.00	0.55, 1.80	0.83	0.43, 1.62	0.90	0.42, 1.90	
	Low quality, low inaccessible, agricultural green							0.55
	Q2	0.86	0.49, 1.52	0.90	0.46, 1.17	1.09	0.48, 2.50	
	Q3	0.76	0.43, 1.35	0.97	0.49, 1.93	1.15	0.47, 2.81	
	Q4	0.74	0.41, 1.31	1.20	0.56, 2.58	1.35	0.50, 3.65	
*Obese*								
	High food, low FEHI, high walkability							0.36
	Q2	1.07	0.53, 2.18	1.31	0.60, 2.82	1.44	0.627, 3.285	
	Q3	1.12	0.56, 2.27	1.10	0.49, 2.48	1.31	0.488, 3.497	
	Q4	1.98*	1.04, 3.78	1.61	0.60, 4.29	1.83	0.555, 5.997	
	Relative food environment advantage							0.13
	Q2	0.60	0.31, 1.16	0.46*	0.22, 0.95	0.50	0.23, 1.07	
	Q3	0.49*	0.25, 0.95	0.43*	0.21, 0.90	0.48	0.21, 1.08	
	Q4	0.53	0.27, 1.05	0.46	0.21, 1.01	0.49	0.21, 1.11	
	Low density and high distance to accessible green							
	Q2	1.08	0.55, 2.10	1.03	0.49, 2.13	1.34	0.61, 2.95	0.88
	Q3	1.15	0.60, 2.25	1.40	0.68, 2.87	1.54	0.74, 3.21	
	Q4	1.05	0.53, 2.07	0.72	0.34, 1.54	1.00	0.43, 2.33	
	Low-quality, agricultural/inaccessible green							0.16
	Q2	1.21	0.60, 2.43	0.92	0.42, 2.03	1.379	0.524, 3.628	
	Q3	1.24	0.62, 2.47	1.14	0.51, 2.56	1.772	0.638, 4.921	
	Q4	1.48	0.76, 2.91	1.51	0.65, 3.55	2.144	0.715, 6.427	

Multivariate model 1 included the individual principal components per model corrected for age, sex, educational level, migration background, SES-WOA score of 4-digit postal code and population density. Multivariate model 2 included all principal components corrected for the aforementioned confounders. The first quartile of the principal components was set as the reference category

*Bold values indicate *p *< 0.05

Univariate results also showed statistically significant higher odds of obesity for patients in higher quartiles of the “*High food*,* low FEHI*,* high walkability*” component (Q4: OR = 1.98, 95% CI [1.04, 3.78]), however, this association was no longer significant in the adjusted models.

### Associations with diet quality

In the linear regression analysis (Table 4), a statistically significant association was observed for the *“Low quality*,* agricultural/inaccessible green”* component. In the fully adjusted model (model 2), participants in the second quartile (Q2) had lower DHD scores compared with those in the lowest quartile (β = −6.00, 95% CI [− 11.58, − 0.41]). This association was not observed for higher quartiles, and no significant trend across quartiles was found (P-trend = 0.37). For the other components no statistically significant associations with the DHD index were identified in either univariate or multivariate analyses.

**Table 4:** Result of univariate and multivariate regression analysis between varimax rotated principal components and total DHD-index score

Rotated Components		Univariate analyses		Multivariate model 1		Multivariate model 2		*P* for trend
		β	95% CI	β	95% CI	β	95% CI	
High food, low FEHI, high walkability	Q2	-0.70	-5.05, 3.66	-1.01	-5.47, 3.45	-2.10	-6.87, 2.68	0.22
	Q3	-0.78	-5.12, 3.55	-1.09	-5.79, 3.62	-3.94	-9.74, 1.87	
	Q4	-1.64	-6.00, 2.71	-0.17	-6.29, 5.95	-3.94	-11.36, 3.48	
Relative food environment advantage	Q2	1.34	-2.96, 5.64	0.55	-3.79, 4.90	-0.04	-4.52, 4.44	0.12
	Q3	1.76	-2.67, 6.19	-0.13	-4.59, 4.34	-0.97	-5.84, 3.90	
	Q4	-0.83	-5.30, 3.64	-3.02	-7.71, 1.65	-3.60	-8.47, 1.27	
Low density and high distance to accessible green	Q2	-0.72	-5.04, 3.61	-1.28	-5.62, 3.06	-1.33	-6.02, 3.36	0.56
	Q3	0.63	-3.70, 4.96	0.11	-4.18, 4.41	-0.33	-4.70, 4.03	
	Q4	-2.05	-6.48, 2.37	-0.64	-5.08, 3.80	-1.78	-6.75, 3.20	
Low-quality, agricultural/inaccessible green	Q2	-4.25	-8.61, 0.10	-4.11	-8.66, 0.45	-6.00*	-11.58, -0.41	0.37
	Q3	-2.38	-6.74, 1.98	-2.29	-7.00, 2.41	-4.87	-10.9, 1.12	
	Q4	-4.24	-8.59, 0.11	-2.81	-7.94, 2.31	-6.23	-12.9, 0.39	

Multivariate model 1 included the individual principal components per model corrected for age, sex, educational level, migration background, SES-WOA score of 4-digit postal code and population density. Multivariate model 2 included all principal components corrected for the aforementioned confounders. The first quartile of the principal components was set as the reference category

*Bold values indicate *p* < 0.05

### Associations with physical activity

The linear regression analyses (Table 5) indicated a significant negative association between the highest quartile of the *“Relative food environment advantage”* component and total minutes of physical activity per week in the univariate analysis (β = −3.85, 95% CI [− 7.44, − 0.26]). This suggests that patients living in neighbourhoods with a relatively higher proportion of healthy food retailers reported fewer minutes of physical activity per week. However, this association was no longer statistically significant after adjustment for individual and neighbourhood-level confounders in models 1 and 2. For the other components, no statistically significant associations were observed in either univariate or multivariate models.

**Table 5:** Result of univariate and multivariate regression analysis between varimax rotated principal components and total minutes of physical activity per week (square root transformed)

		Univariate analyses		Multivariate model 1		Multivariate model 2		*P* for trend
		β	95% CI	β	95% CI	β	95% CI	
High food, low FEHI, high walkability	Q2	-0.29	-3.77, 3.18	-0.46	-3.90, 2.98	-0.60	-4.27, 3.08	0.23
	Q3	-2.55	-6.00, 0.90	0.08	-3.53, 3.69	0.60	-3.86, 5.05	
	Q4	1.25	-2.23, 4.73	3.55	-1.18, 8.28	4.43	-1.33, 10.19	
Relative food environment advantage	Q2	-1.75	-5.18, 1.68	-0.20	-3.55, 3.14	0.23	-3.21, 3.66	0.80
	Q3	-1.96	-5.48, 1.56	-0.04	-3.48, 3.41	1.22	-2.50, 4.95	
	Q4	-3.85*	-7.44, -0.26	-1.32	-4.97, 2.33	-0.61	-4.38, 3.16	
Low density and high distance to accessible green	Q2	-1.93	-5.40, 1.55	-2.23	-5.59, 1.13	-1.56	-5.18, 2.07	0.52
	Q3	-1.08	-4.54, 2.39	-1.40	-4.69, 1.90	-1.12	-4.47, 2.23	
	Q4	-0.96	-4.52, 2.59	-2.45	-5.87, 0.97	-1.37	-5.19, 2.45	
Low-quality, agricultural/inaccessible green	Q2	0.36	-3.14, 3.85	-0.94	-4.45, 2.57	-0.83	-5.13, 3.48	0.26
	Q3	0.72	-2.78, 4.21	-2.31	-5.93, 1.30	-1.76	-6.39, 2.87	
	Q4	3.19	-0.28, 6.66	1.80	-2.15, 5.74	2.32	-2.79, 7.42	

Multivariate model 1 included the individual principal components per model corrected for age, sex, educational level, migration background, SES-WOA score of 4-digit postal code and population density. Multivariate model 2 included all principal components corrected for the aforementioned confounders. The first quartile of the principal components was set as the reference category

*Bold values indicate *p* < 0.05

### Additional outcomes and sensitivity analyses

Analyses of additional health behaviours are presented in Additional File 2.

Analysis of the fruit and vegetable subcomponent of the DHD revealed a significant association with the “*Relative food environment advantage”* component. In both multivariate models, participants in the highest quartile had significantly lower fruit and vegetable diet quality scores compared with those in the lowest quartile (model 2: β=-1.70, 95% CI [-3.10, -0.31], P-trend = 0.01). No significant associations were observed between fruit and vegetable intake and the other three PCs (Table A4a).

For the unhealthy food choices subcomponent, the “*Low density and high distance to accessible green*” component showed a significant association in univariate and model 1 (model 1: OR = 0.56, 95% CI [0.33, 0.96]) analyses. However, the association was no longer statistically significant after full adjustment in model 2. No other PCs showed a significant association with unhealthy food choices (Table A4b).

Binary analysis of meeting the physical activity guideline (≥ 150 min of moderate to vigorous activity per week) revealed a significant univariate association for the “*Low quality*,* agricultural/inaccessible green*” component. Participants in Q3 had higher odds of meeting the guidelines compared with Q1 (OR = 1.72, 95% CI [1.01, 2.90]). However, this association was no longer significant after adjustment for confounders in models 1 and 2. No other PCs showed significant associations with meeting the physical activity guidelines in any model (Table A4c).

Effect modification for SES-WOA score and the PCs was tested by including an interaction term in model 2 of the multinomial logistic regression model for weight status and the linear models for total DHD-score and total minutes of weekly physical activity (Table A4d-f). In none of the models was the interaction term statistically significant, except for the interaction between the “*Relative food environment advantage*” component and neighbourhood socioeconomic position for obesity (*P* < 0.02), though not for overweight. However, the neighbourhood socioeconomic position main effect showed an extremely low OR likely reflecting model instability due to sparse data (only 11 obese participants in highest tertile of SES-WOA).

## Discussion

This study set out to do two things: to characterise empirically how a broad set of neighbourhood environmental indicators co-occur in a dense Dutch urban setting, and to explore whether the resulting patterns associate with weight status, diet quality and physical activity in a primary care population at high cardiovascular risk. The first aim produced what we consider the more substantive contribution: a clear demonstration of how different neighbourhood features covary and that, within a single dense city, absolute measures of the food environment cannot be meaningfully separated from population density, even after restricting to the most highly urbanised neighbourhoods. The second aim produced mostly null or inconsistent associations, with one component (capturing the relative composition of healthy versus unhealthy food retailers) showing modest and somewhat paradoxical signals. We discuss the implications of each in turn.

### Population density and the food environment

An important finding was the very strong positive correlation between food retailer density and population density. Crucially, this strong correlation persisted even within the “very strongly urban” category (> 2,500 addresses per km²). Previous research in compact Dutch urban settings has shown that population density itself is associated with multiple health-relevant pathways, underscoring its role as a broader structuring factor of urban environments [41]. Beyond these physical and exposure-mediated consequences, population density between neighbourhoods may also reflect social and historical processes (such as residential self-selection, life stage migration, shared values and social housing allocation), thus associations between density-related environmental exposures and health can additionally reflect reverse pathways [42, 43]. 

Our approach of adjusting for continuous population density addresses this limitation, but introduces a different concern: population density correlated very strongly with PC1 (*r* = 0.86), meaning that adjusting for it may have removed most of the variation this component captures. This raises a fundamental issue: failing to adjust for population density risks confounding, yet adjusting for it may obscure real associations between the food environment and health outcomes that operate precisely through the urban density gradient. The consequence of this limitation is illustrated in our results: the PC1–obesity association attenuated after adjustment for contextual neighbourhood variables, a pattern that is equally compatible with three different stories: no effect, a real effect masked by collinearity, and a real indirect effect operating through the density gradient that adjustment over-controls. Our regression cannot distinguish between them.

This observation has implications for the broader food environment literature: simply adjusting for urbanicity category might not adequately control for population density-related confounding. Our data show that the residual density variation within even the highest category of population density is substantially correlated with retail density. Adjustment solely for urbanicity category therefore might not address this sufficiently, and effect estimates from such designs should be interpreted with care.

Two design choices can help partially mitigate but not resolve this entanglement. The first, and arguably the strongest, is to use longitudinal variation when retailer composition changes within a neighbourhood, such as through natural experiments. The second is to exploit cross-city variation in the density–environment coupling itself: in some urban contexts the relationship between population density and food-retail density is weaker or qualitatively different, and including such heterogeneous settings can help identify off-diagonal cases [44, 45]. A third route is to use relative measures that are mathematically decoupled from density, as described below.

### Relative food environment measures are mathematically separable from density

Relative and absolute food environment measures loaded on separate principal components and showed weak correlations with each other. This independence has been noted previously by Pinho et al. [27] and indicates these measures capture fundamentally different dimensions of the food environment. While PC2 is primarily defined by strong loadings on relative measures, it was accompanied by moderate negative loadings on absolute non-core retailer density but not core retailer density. Neighbourhoods scoring high on this component thus achieve their relative advantage primarily through fewer unhealthy retailers, not through greater availability of healthy retailers.

This separation from population density is, in part, a mathematical consequence of the ratio structure: when both core and non-core retailer counts scale approximately proportionally with population density, the ratio between them is approximately invariant under density variation. This implies that associations for PC2 with health outcomes cannot be attributed to residual density effects.

However, even though calls have been made to use relative measures more widely [27, 46, 47], this independence also presents interpretive challenges. Identical relative scores can represent fundamentally different food environments depending on absolute availability. For example, a neighbourhood with 2 supermarkets and 1 fast-food outlet (67% healthy) scores better than one with 20 supermarkets and 30 fast-food outlets (40% healthy), despite the latter offering far more absolute healthy options. Moreover, as previously described [48], ratio measures can be volatile in low-density areas, where a single retailer opening or closing can shift the ratio by 10% points, while the same change in a dense area produces less than a 1-percentage-point shift. Future research should consider: restricting analyses to areas exceeding minimum retail density thresholds [48] and/or stratifying by absolute retailer density (not urbanicity) to test whether associations are consistent across contexts.

### “Relative food environment advantage" association with weight status

The PC2 (“*Relative food environment advantage*” component) results provide one of the more consistent signals in this study. This is consistent with a systematic review by Cobb. et al. which noted that studies utilizing relative measures are more likely to see significant and expected results with weight status than absolute measures [3]. This component showed consistent associations with lower odds of being overweight across all models for the third quartile (univariate, multivariate model 1, and fully adjusted model 2). Similar protective associations were observed for obesity in univariate and model 1 analyses, though these attenuated in the fully adjusted model. However, no clear dose-response pattern emerged (P-trend = 0.19), suggesting potential threshold effects or that these associations may be influenced by other unmeasured neighbourhood characteristics. This modest finding is also complicated by a counterintuitive pattern: higher levels of this same component were associated with lower fruit and vegetable intake in the highest quartile (including a significant trend). A systematic review by Turner et al., studying the association between the retail environment and fruit and vegetable consumption, found mixed results [49]. However, most of the included studies focused on absolute measures. Previous studies investigating the association between relative measures and fruit/vegetable intake in the general population found results in the opposite direction, i.e. reporting that individuals living in areas with a higher proportion of healthier food stores reported greater fruit and vegetable consumption [46, 47, 50]. The loading structure in our sample might offer a mechanistic, although speculative, interpretation of this pattern: because it captures relative advantage primarily through reduced exposure to non-core retailers rather than increased absolute availability of healthier alternatives, residents in higher PC2 neighbourhoods are less surrounded by unhealthy retailers (potentially explaining the protective association with weight status) but their absolute access to healthy alternatives is not elevated (providing no mechanism by which fruit and vegetable diet consumption would be expected to increase).

Also, we found no evidence that associations between neighbourhood characteristics and health outcomes varied by neighbourhood socioeconomic position, except for a significant interaction between the “Relative food environment advantage” component and neighbourhood socioeconomic position for obesity, suggesting that food environment characteristics may be more strongly associated with obesity in neighbourhoods with a lower socioeconomic position. This finding is consistent with recent Dutch evidence [51]. However, model instability was observed due to low numbers within strata, necessitating replication in larger samples before drawing substantive conclusions.

### Green space

Our analysis revealed substantial heterogeneity in green space measures. NDVI and other, mostly agricultural, green space loaded together (PC4) but showed weak correlations with accessible green spaces (PC3), reflecting that these measures capture fundamentally different constructs. NDVI primarily captures vegetation exposure correlating with larger agricultural land and might be more relevant to air quality and heat mitigation, while accessible green spaces capture opportunities for physical activity and social interaction [52]. Besides, green space showed expected inverse relationships with population density. Relationships with neighbourhood socioeconomic position were weaker and this finding aligns with previous Dutch research showing that socioeconomic differences in greenness are smaller in highly urbanised municipalities [53]. 

The few significant findings between PC3 and PC4 with health outcomes failed to show consistent patterns or dose-response relationships and/or independent associations from confounding.

### Scale and spatial considerations

Our data showed high correlations between environmental characteristics at 300 m and 300–1000 m scales. Neighbourhoods with high retail or green space density immediately around residences (300 m) generally also have high density in their extended surroundings (300–1000 m donut buffer). At the address level, however, cross-scale correlations were considerably lower, and the correlation of each indicator with population density depended strongly on buffer size (donut indicators kept being strongly correlated with population density but 300 m indicators showed lower correlation on address level). This phenomenon is known as modifiable areal unit problem [54] and indicates that postal-code averaging smooths variation across immediate and extended neighbourhood. For example, a single address can have a very different food environment in the 300 m buffer than in the donut buffer, but summing all 300 m buffers across a postal code averages out this within-area variation. For studies with access to patient-level address data, smaller buffers may partially decouple the food environment exposure from population density.

An important exception emerged. Relative food environment measures showed lower cross-scale correlations (ρ ≈ 0.6), suggesting that the relative composition of healthy versus unhealthy food retailers can vary between immediate and extended neighbourhoods. This has implications for mechanism: if the relative food environment primarily influences health through daily shopping patterns, immediate neighbourhood exposure (300 m, walkable distance) may matter more than extended area composition. Conversely, if mechanisms operate through neighbourhood norms or perceived availability as previously proposed [55–57], extended area characteristics might be more relevant.

### Implications for high-risk populations

The second aim of this study was to investigate whether environmental patterns are associated with weight and lifestyle behaviours in a primary care high-risk population. Only the “*Relative food environment advantage*” showed a more consistent association pattern with weight but also counter-intuitively with fruit and vegetable diet quality.

Several considerations should be taken into account. First, the identification problem discussed above for PC1 is a feature of the setting, not the population, and applies equally to general-population studies. Second, individuals already at high cardiovascular risk may exhibit reduced sensitivity to environmental influences, as long-standing dietary habits or overriding determinants, such as specific stressors or health status may play a more dominant role in shaping diet. Third, and partly counterbalancing this, high-cardiovascular-risk patients tend to be older (mean age 65.6 in our sample) and may have spatially smaller activity spaces than working-age adults, spending a larger proportion of their daily time within their residential neighbourhood. Residential exposure assessment may therefore be a more representative proxy for total environmental exposure in this group than in younger samples. Fourth, high-risk patients in our study were engaged with healthcare providers who provided dietary guidance as part of standard Dutch primary care protocols, potentially creating alternative pathways to healthy behaviours that are independent of neighbourhood characteristics. Fifth, residential self-selection, which may be particularly relevant in a high-risk group with established health concerns, could operate in either direction. Further large-scale confirmatory research and different designs are therefore needed to explore these findings more robustly.

### Strengths and limitations

A major strength of this study was the comprehensive data-driven assessment of the neighbourhood environment, including 23 indicators across food environment, green space, and walkability domains at two spatial scales. By applying PCA for dimensionality reduction, we revealed distinct environmental patterns and their intercorrelations, providing insights into how neighbourhood characteristics cluster in dense urban settings. Furthermore, to our knowledge, this is the first study that investigates the association between neighbourhood characteristics and BMI and lifestyle behaviour in a primary care population at high risk for CVD. While the identified environmental patterns likely reflect general characteristics of the urban landscape applicable across populations, the associations with health behaviours were examined specifically in this high-risk group. Whether environmental patterns play a similar role in the general population remains an open question. As previously mentioned, high-risk populations may differ from the general populations in important ways which could modify how they interact with their neighbourhood environment. Nonetheless, studying this population offers preliminary insights relevant for population health management and risk stratification in primary care settings.

Several limitations should be noted. First, the cross-sectional exploratory design in a single highly urbanised area precludes causal inference and limits generalisability to less urbanised contexts. The large number of statistical tests across multiple outcomes and components increases the probability of chance findings; no correction for multiple testing was applied, consistent with the exploratory aims, but individual significant associations should be interpreted accordingly.

Second, exposure was operationalised at the 4-digit postal code level due to privacy considerations; as shown in our address-level analyses, this aggregation reduces sensitivity to fine-grained environmental variation. Several environmental factors potentially relevant for cardiometabolic health (most notably air pollution and traffic-related noise) were not measured. These typically co-occur with dense built environments, meaning that part of the PC1 attenuation under density adjustment may reflect uncontrolled exposure to correlated stressors rather than density per se. Outcomes were additionally assessed by self-report, which may introduce socially desirable responding and underestimate observed associations.

Third, only individual sociodemographic confounders were included in the models. We did not adjust for clinical confounders such as 10-year CVD risk score, medication use, or specific comorbidities. This was a deliberate choice for three reasons: sample size constraints limited the number of covariates we could reliably include; patients with diabetes or established cardiovascular disease were already excluded from the cohort, narrowing the relevance of these comorbidities as confounders; and clinical risk factors and medication use are plausibly downstream of, rather than antecedent to, the lifestyle behaviours and weight status, so adjusting for them would risk overadjustment for variables on the causal pathway. Residual between-patient variation in disease severity and treatment intensity could nonetheless influence lifestyle behaviours through pathways unrelated to environmental exposure.

Finally, PCA involves an inherent trade-off: it captures co-occurrence and reduces multicollinearity efficiently, but composite scores obscure the contribution of specific attributes within each pattern. The PCA was additionally conducted on the postal codes of participants rather than on all neighbourhoods in The Hague; although the included postal codes cover 79% of those in The Hague and adjacent municipalities, capturing substantial environmental and sociodemographic variation, the identified patterns may not fully represent the entire urban area.

## Conclusions

This study characterised how a broad set of food environment, green space and walkability indicators co-occur within a single dense Dutch urban area, and examined whether the resulting neighbourhood patterns relate to weight status, diet quality and physical activity in primary-care patients at high cardiovascular risk. PCA produced four interpretable patterns. The dominant pattern combined retail density, walkability and limited green space, and was tightly coupled to population density, even within the most highly urbanised stratum. Cross-sectional designs in such settings cannot easily identify independent effects of these environmental factors on health through statistical adjustment alone.

Of the identified patterns, only one, the “*Relative food environment advantage*” pattern (which was not entangled with population density), showed a more robust association with a lower odds of being overweight in a high-risk primary care sample. However, this association was modest, showed no consistent dose–response, and was accompanied by a paradoxical association with lower fruit and vegetable dietary quality. No robust associations were observed for physical activity or other dietary outcomes. There was some indication that the relative food environment may be more strongly associated with obesity in neighbourhoods with lower socioeconomic position, suggesting possible effect modification that warrants further exploration.

These findings highlight the value of examining neighbourhood characteristics as interrelated patterns rather than isolated exposures, while underscoring the interpretive and analytical difficulties this approach reveals rather than resolves. Larger, confirmatory studies, taking into account the methodological difficulties discussed, are needed to investigate the preliminary associations with health outcomes in high-risk populations.

## Supplementary Information

Supplementary Material 1.

Supplementary Material 2.

## Data Availability

Data from the Healthy Heart study may be available from the corresponding author upon reasonable request, subject to ethics approval (Leiden UMC, P17.079). Environmental data were obtained from GECCO (walkability index, available upon request via [https://www.gecco.nl]) and Locatus (commercial data, available under licence). Green space data from the Dutch Land Use Database (CBS) and Landsat 8 satellite imagery (USGS) are publicly available.

## Abbreviations

BAG: Basisregistratie Adressen en Gebouwen (Registration of Addresses and Buildings)
BMI: Body mass index
CBS: Centraal Bureau voor de Statistiek (Statistics Netherlands)
CI: Confidence interval
CVD: Cardiovascular disease
DHD-FFQ: Dutch Healthy Diet Food Frequency Questionnaire
DHD-index: Dutch Healthy Diet index
FEHI: Food Environment Healthiness Index
GECCO: Geoscience and Health Cohort Consortium
GP: General practitioner
ICPC: International Classification of Primary Care
IQR: Interquartile range
KMO: Kaiser-Meyer-Olkin
MRFEI: Modified Retail Food Environment Index
NDVI: Normalised Difference Vegetation Index
NHG: Nederlands Huisartsen Genootschap (Dutch College of General Practitioners)
PC: Principal component
PCA: Principal component analysis
SD: Standard deviation
SEP: Socioeconomic position
SES-WOA: Sociaaleconomische status op basis van welvaart, opleidingsniveau en arbeidspositie
SPSS: Statistical Package for the Social Sciences
SQUASH: Short Questionnaire to Assess Health-enhancing physical activity
VIF: Variance inflation factor
