# Supplementary material for: Identifying co-occurring neighbourhood environmental patterns and their association with health behaviours in a Dutch urban population at high cardiometabolic risk
Source: BMC Public Health. 2026 Jul 7;26:2692. doi: 10.1186/s12889-026-28175-6 (PMC13621774; doi:10.1186/s12889-026-28175-6)
Supplement: Supplementary file 1 [file 12889_2026_28175_MOESM1_ESM.docx]

# Additional File 1: Extended Methodological Details

This file provides detailed descriptions of the environmental indicators, geospatial analysis procedures, data preparation steps and health outcome definitions used in the main manuscript.

## A1. Environmental indicator definitions and geospatial methods

***A1.1 Spatial units and buffer creation***

Environmental characterization was conducted for 73 four-digit postal codes where study participants resided. All geospatial analyses were performed using QGIS version 3.18.3. Environmental indicators were first calculated at the address level using residential address locations from the national Registration of Addresses and Buildings (BAG) [19], and then averaged at the postal code level.

Indicators were assessed within two spatial scales around each residential address: a 300-metre Euclidean buffer (immediate neighbourhood) and a 300–1000-metre donut-shaped buffer (extended neighbourhood). The 300-metre buffers were created using the standard buffer function in QGIS. The donut buffers were generated by computing the geometric difference between a 1000-metre outer buffer and a 300-metre inner buffer using the following pyQGIS script:

from qgis.core import (

QgsVectorLayer,

QgsProject,

QgsField,

QgsFeature,

QgsGeometry,

QgsWkbTypes

)

\# Define input and output layer names

input_layer_name = 'houses' # Change this to match the name of your point layer

output_layer_name = 'donut_layer'

\# Get reference to the input point layer

input_layer = QgsProject.instance().mapLayersByName(input_layer_name)[0]

\# Define buffer distances

inner_distance = 300 # in meters

outer_distance = 1000 # in meters

\# Create new memory layer for donut features

donut_layer = QgsVectorLayer('Polygon?crs=' + input_layer.crs().authid(), output_layer_name, 'memory')

provider = donut_layer.dataProvider()

\# Add attribute fields to the donut layer

fields = input_layer.fields()

provider.addAttributes(fields)

donut_layer.updateFields()

for feature in input_layer.getFeatures():

outer_buffer = feature.geometry().buffer(outer_distance, 30)

inner_buffer = feature.geometry().buffer(inner_distance, 30)

donut_geometry = outer_buffer.difference(inner_buffer)

if donut_geometry.isGeosValid(): # Check if the geometry is valid

donut_feature = QgsFeature(fields)

donut_feature.setGeometry(donut_geometry)

donut_feature.setAttributes(feature.attributes())

provider.addFeature(donut_feature)

donut_layer.commitChanges()

\# Add the donut layer to the map

QgsProject.instance().addMapLayer(donut_layer)

***A1.2 Food environment indicators***

Food environment data, published in 2019, were obtained from Locatus, a company that collects information on business locations through regular field audits [20]. Food retailers were classified following previous examples [21]:

- **Core food retailers** (healthier options): vegetable/fruit shops, nut shops, organic shops, fish shops, oriental supermarkets, supermarkets, and mini-supermarkets.
- **Non-core food retailers** (less healthy options): fast food outlets, delivery/take-away outlets, grillrooms/kebab shops, ice cream shops, sweet shops, chocolate shops, cake/muffin shops, pancake shops, crêpe/donut shops, and flan shops ("vlaaien").

**Absolute measures**

**Density of core and non-core food retailers:** Count of retailers within each buffer zone, calculated using the ‘count points in polygons’ function in QGIS.

**Food Environment Healthiness Index (FEHI) score:** The FEHI, developed by Poelman et al. [21] using a Delphi procedure, assigns a health score ranging from −5 (more unhealthy) to +5 (more healthy) to individual food retailers in the Locatus database, accounting for variation in healthiness within the core and non-core categories. The FEHI score within each buffer was calculated as the sum of individual retailer FEHI scores, using ‘count points in polygons’ with the FEHI score as weight field.

**Spatial accessibility to core and non-core retailers**. Sum of inverse Euclidean distances to all core (or non-core) retailers within the buffer. This measure accounts for the fact that retailers closer to home may exert greater influence than those farther away within the buffer zone. The calculation was performed using the QGIS field calculator:

array_sum(

array_foreach (

overlay_nearest ('restaurant_layer', $geometry, max_distance:=(insert buffersize, e.g. 300m or 1000m), limit:=-1),

1 / distance ($geometry, @element)

)

)

**Relative measures**

**Modified Retail Food Environment Index (MRFEI) [22, 23]:** Percentage of core food retailers out of all food retailers within the buffer, calculated as:

A score of one was added to the number of healthy and unhealthy food retailers to avoid division by zero. A higher percentage means a higher proportion of healthy food outlets compared to less healthy options.

**Relative spatial accessibility:** Ratio of spatial access to core food retailers out of spatial access to all food retailers, calculated as:

A score of one was added to the spatial access scores for healthy and unhealthy food retailers to avoid division by zero. A higher ratio indicates greater spatial access to healthy relative to unhealthy food outlets.

***A1.3 Green space indicators***

Green space indicators were assessed using Dutch Land Use Database data (Bestand Bodemgebruik, 2017) [24] and Landsat 8 satellite imagery accessed through USGS EarthExplorer [25].

**Percentage of accessible green spaces:** Proportion of buffer area covered by green spaces that residents can freely enter. Accessible green spaces were defined as areas with the following labels in the CBS Land Use dataset: park and ornamental grounds, day recreation area, holiday recreation area, forest, dry natural open land, and wet natural open land. The area of green space within each buffer was determined using the intersect function (between the buffer layer and green space layer), followed by the dissolve function (based on buffer ID). Area was calculated using the $area function (in m²) in the field calculator. To prevent loss of residential addresses without any green space in the buffer, the area variable from the dissolved layer was merged with the original buffer layer and empty cells were set to zero. Percentage of green space within the buffer was then calculated as: (green space area / total buffer area) × 100.

**Percentage of other green spaces**: Proportion of buffer area covered by non-freely accessible green spaces, defined as areas labelled sports area, allotment garden, or other agricultural land in the CBS Land Use dataset. Greenhouses were excluded. The same geospatial procedure as described above was applied.

**Network distance to nearest accessible green space:** Shortest walking distance (in meters) to the nearest entrance of an accessible green space, calculated via the road network. Green space polygons were transformed to lines, and the intersection of lines tool in QGIS was used to identify points where roads intersected with green space boundaries; these points served as access points. The QNEAT3 plugin was used to calculate the origin-destination cost matrix from residential addresses to access points. The nearest access point per origin was identified using the following SQL query:

SELECT origin_id, destination_id, MIN(total_cost) AS shortest_distance, geometry

FROM input1

GROUP BY origin_id

Due to computational limitations, network distance was calculated for a 5% sample of residential addresses (minimum 50 metres apart) within each postal code.

**Normalised Difference Vegetation Index (NDVI):** Average NDVI value within the buffer, calculated from Landsat 8 Band 4 (red) and Band 5 (near-infrared) data using the raster calculator: NDVI = (Band 5 – Band 4) / (Band 5 + Band 4). Higher values indicate more and healthier vegetation. Mean NDVI per buffer was calculated using zonal statistics. NDVI scores were not adjusted for water bodies.

## A2. Data preparation for principal component analysis

***A2.1 Box-Cox transformation***

Prior to PCA, all environmental variables were Box-Cox transformed to approximate a normal distribution and to diminish the effect of natural outliers. The Box-Cox transformation is defined as:

where y is the original data and λ is the transformation parameter (range: −5 to 5).

Because the Box-Cox transformation cannot handle zero or negative values, a constant of 1 was added to all variables before transformation. Across all postal codes, the average FEHI scores were zero or negative; therefore, the absolute value was computed before adding the constant. As a result, higher transformed FEHI scores indicate a higher concentration of unhealthy retailers (i.e., the interpretation is inverted compared to the original FEHI scores). Throughout the manuscript, the transformed variable is referred to as ‘inverse FEHI’.

After transformation, all variables were z-standardised to ensure a common scale.

## A3. Health outcome variable definitions

***A3.1 Diet quality***

Diet quality was assessed using the Dutch Healthy Diet index (DHD-index) [32, 33] based on the Dutch Healthy Diet Food Frequency Questionnaire (DHD-FFQ). The DHD-index has been recognised as a reliable tool for ranking participants based on diet quality. The total score ranges from 0 to 160, covering 16 components (0–10 per component), with higher scores indicating better adherence to the 2015 Dutch Guidelines for a Healthy Diet.

***A3.2 Physical activity***

Physical activity was measured using the validated SQUASH questionnaire [34], which estimates physical activity across leisure time, commuting, work, and household domains in minutes per week.

***A3.3 Body weight status***

BMI (kg/m²) was calculated from self-reported height and weight and categorised based on age, following the recommendations of the Dutch Nutrition Centre ("Voedingscentrum") [35]:

- Patients aged 19–69 years: healthy weight (BMI ≥18.5 and <25), overweight (BMI ≥25 and <30), obese (BMI ≥30)
- Patients aged ≥70 years: healthy weight (BMI ≥22 and <28), overweight (BMI ≥28 and <30), obese (BMI ≥30)

**References**

*All reference numbers in this file correspond to the reference list of the main manuscript.*
