# Supplementary material for: Identifying co-occurring neighbourhood environmental patterns and their association with health behaviours in a Dutch urban population at high cardiometabolic risk
Source: BMC Public Health. 2026 Jul 7;26:2692. doi: 10.1186/s12889-026-28175-6 (PMC13621774; doi:10.1186/s12889-026-28175-6)
Supplement: Supplementary file 2 [file 12889_2026_28175_MOESM2_ESM.docx]

# Additional File 2: Results of additional analyses

## A2.1 Results of the correlation analysis between environmental indicators and contextual neighbourhood variables.

**Figure A1a. Correlation table between environmental indicators and contextual neighbourhood variables at PC4 level.**

*For definition of the environmental indicators see Table 1. Pop_density (population density) defined as addresses per km^2^. SES: Measure of socioeconomic status at the local level, based on a combination of data regarding financial prosperity, educational level and recent employment history of the households. [30]

**Figure A1b. Correlation table between environmental indicators and contextual neighbourhood variables at address level.**

*For definition of the environmental indicators see Table 1. Pop_density (population density) defined as addresses per km^2^. SES: Measure of socioeconomic status at the local level, based on a combination of data regarding financial prosperity, educational level and recent employment history of the households. [30]

**Figure A1c. Correlation table between environmental indicators and contextual neighbourhood variables at address level within very highly urban areas.**

## A2.2 Results of the correlation analysis between principal components and contextual neighbourhood variables

**Figure A2. Correlation table of correlation between principal components and contextual neighbourhood variables**

*For definition of the environmental indicators see Table 1. Pop_density (population density) defined as addresses per km^2^. SES: Measure of socioeconomic status at the local level, based on a combination of data regarding financial prosperity, educational level and recent employment history of the households. [30]

## A2.3 Explained variance of principal components

***Table A3. Explained variance of varimax rotated principal components***

|  | **PC1** | **PC2** | **PC3** | **PC4** |
| --- | --- | --- | --- | --- |
| Variance | 9.621 | 4.148 | 2.321 | 3.838 |
| Proportional variance | 0.418 | 0.180 | 0.101 | 0.167 |
| Cumulative variance | 0.418 | 0.599 | 0.700 | 0.866 |

## A2.4 Additional analyses

The results of the additional analyses are presented below. The following outcomes were included:

- Fruit and vegetable diet quality: a subcomponent of the DHD-index was calculated as the sum of the fruit and vegetable scores (range: 0-20).
- Unhealthy choices: Another DHD-index subcomponent (range: 0-10) was dichotomized as ≥5 (more healthy) or <5 (more unhealthy), given the skewed distribution with many participants scoring either 0 or 10.
- Meeting the physical activity guideline: a binary outcome was derived from the SQUASH data indicating whether participants met the guideline of ≥150 minutes of moderate to vigorous physical activity per week.
- Effect modification by neighbourhood SES: Because the impact of the built environment may differ across different socioeconomic backgrounds as poor living environments are also more common in disadvantaged neighbourhoods with multiple health problems, effect modification between the principal components and the socioeconomic status of the neighbourhood was tested. An interaction term between the ordinal principal component and the continuous SES-WOA score was calculated and added to the main outcome models as a continuous variable. Effect modification was only tested in model 2.

***Table A4a. Result of univariate and multivariate regression analysis between varimax rotated principal components and DHD-subcomponent fruit and vegetable***

| Rotated Components |  | Univariate analyses |  | Multivariate model 1 |  | Multivariate model 2 |  |  |
| --- | --- | --- | --- | --- | --- | --- | --- | --- |
|  |  | β | 95% CI | β | 95% CI | β | 95% CI | P for trend |
| High food, low FEHI, high walkability | Q2  Q3  Q4 | 0.38  -0.09  -0.30 | -0.88, 1.63  -1.34, 1.16  -1.55, 0.95 | 0.29  -0.19  -0.15 | -1.05, 1.63  -1.56, 1.18  -1.50, 1.20 | 0.19  -0.70  -0.70 | -1.17, 1.55  -2.18, 0.77  -2.10, 0.70 | 0.24 |
| Relative food environment advantage | Q2  Q3  Q4 | 0.24  0.21  -0.89 | -0.99, 1.47  -1.05, 1.48  -2.17, 0.39 | 0.07  -0.33  **-1.51*** | -1.19, 1.33  -1.62, 0.95  -2.86, -0.16 | -0.03  -0.57  **-1.70*** | -1.30, 1.24  -1.94, 0.80  -3.10, -0.31 | **0.01*** |
| Low density and high distance to accessible green | Q2  Q3  Q4 | -0.48  -0.01  -0.55 | -1.72, 0.77  -1.25, 1.23  -1.82, 0.72 | -0.64  -0.29  -0.27 | -1.89, 0.62  -1.53, 0.96  -1.59, 1.05 | -0.40  -0.24  -0.14 | -1.74, 0.95  -1.49, 1.01  -1.47, 1.19 | 0.87 |
| Low-quality, agricultural/inaccessible green | Q2  Q3  Q4 | -0.36  -0.21  -0.48 | -1.62, 0.89  -1.46, 1.05  -1.74, 0.77 | -0.67  -0.53  -0.49 | -2.01, 0.67  -1.89, 0.82  -1.83, 0.86 | -0.36  -0.37  -0.51 | -1.78, 1.06  -1.78, 1.05  -1.86, 0.83 | 0.52 |

**p<0.05. Multivariate model 1 included the individual principal components per model corrected for age, sex, educational level, migration background, SES-WOA score of the 4-digit postal code and population density. Multivariate model 2 included all principal components corrected for the aforementioned confounders.*

***Table A4b. Result of univariate and multivariate logistic regression analysis between varimax rotated principal components and DHD-subcomponent unhealthy choices***

| Rotated Components |  | Univariate analyses |  | Multivariate model 1 |  | Multivariate model 2 |  |  |
| --- | --- | --- | --- | --- | --- | --- | --- | --- |
|  |  | OR | 95% CI | OR | 95% CI | OR | 95% CI | P for trend |
| High food, low FEHI, high walkability | Q2  Q3  Q4 | 1.07  0.77  0.79 | 0.64, 1.77  0.46, 1.27  0.48, 1.32 | 1.07  0.79  0.66 | 0.62, 1.83  0.45, 1.40  0.31, 1.39 | 0.94  0.69  0.70 | 0.53, 1.67  0.37, 1.28  0.39, 1.26 | 0.17 |
| Relative food environment advantage | Q2  Q3  Q4 | 0.86  0.86  0.79 | 0.52, 1.41  0.51, 1.43  0.47, 1.33 | 0.92  0.85  0.73 | 0.55, 1.16  0.50, 1.46  0.41, 1.30 | 0.87  0.80  0.67 | 0.51, 1.48  0.45, 1.42  0.37, 1.21 | 0.17 |
| Low density and high distance to accessible green | Q2  Q3  Q4 | 0.74  0.69  **0.58*** | 0.45, 1.23  0.41, 1.13  0.34, 0.97 | 0.76  0.68  **0.56*** | 0.45, 1.29  0.41, 1.15  0.33, 0.96 | 0.76  0.73  0.59 | 0.43, 1.32  0.43, 1.23  0.34, 1.04 | 0.07 |
| Low-quality, agricultural/inaccessible green | Q2  Q3  Q4 | 1.19  1.43  1.19 | 0.71, 1.98  0.86, 2.39  0.71, 1.98 | 1.19  1.36  1.25 | 0.68, 2.06  0.77, 2.41  0.67, 2.33 | 1.07  1.21  1.05 | 0.59, 1.95  0.67, 2.20  0.59, 1.86 | 0.80 |

**p<0.05. Multivariate model 1 included the individual principal components per model corrected for age, sex, educational level, migration background, SES-WOA score of the 4 digit postal code and level of urbanicity. Multivariate model 2 included all principal components corrected for the aforementioned confounders.*

***Table A4c. Result of univariate and multivariate logistic regression analysis between varimax rotated principal components and meeting the physical activity guideline.***

| Rotated Components |  | Univariate analyses |  | Multivariate model 1 |  | Multivariate model 2 |  |  |
| --- | --- | --- | --- | --- | --- | --- | --- | --- |
|  |  | OR | 95% CI | OR | 95% CI | OR | 95% CI | P for trend |
| High food, low FEHI, high walkability | Q2  Q3  Q4 | 0.92  0.68  0.93 | 0.55, 1.54  0.40, 1.16  0.55, 1.56 | 0.78  0.79  0.89 | 0.45, 1.37  0.44, 1.44  0.41, 1.95 | 0.77  0.87  1.15 | 0.43, 1.39  0.45, 1.67  0.63, 2.10 | 0.62 |
| Relative food environment advantage | Q2  Q3  Q4 | 0.71  1.08  0.78 | 0.42, 1.20  0.64, 1.82  0.45, 1.33 | 0.91  1.41  1.10 | 0.52, 1.57  0.81, 2.45  0.61, 1.99 | 0.93  1.52  1.13 | 0.54, 1.62  0.84, 2.75  0.61, 2.09 | 0.48 |
| Low density and high distance to accessible green | Q2  Q3  Q4 | 0.84  0.77  0.93 | 0.50, 1.41  0.46, 1.30  0.55, 1.58 | 1.00  0.71  0.97 | 0.58, 1.75  0.41, 1.22  0.55, 1.70 | 0.98  0.73  1.02 | 0.54, 1.77  0.42, 1.26  0.57, 1.83 | 0.87 |
| Low-quality, agricultural/inaccessible green | Q2  Q3  Q4 | 1.04  **1.72***  1.30 | 0.61, 1.78  1.01, 2.90  0.77, 2.20 | 1.05  1.32  1.07 | 0.58, 1.89  0.73, 2.37  0.56, 2.04 | 1.06  1.40  1.20 | 0.56. 2.00  0.76. 2.61  0.66. 2.18 | 0.39 |

**p<0.05. Multivariate model 1 included the individual principal components per model corrected for age, sex, educational level, migration background, SES-WOA score of the 4 digit postal code and population density. Multivariate model 2 included all principal components corrected for the aforementioned confounders.*

***Table A4d. Interaction Effects of Socioeconomic Status and Principal Component on weight status***

| Weight status | Variables  *Quartiles* | Multivariate model 2 |  |
| --- | --- | --- | --- |
| *Overweight* |  | *OR* | *95% CI* |
|  | High food, low FEHI, high walkability  Q2  Q3  Q4  Socioeconomic status^1^  Socioeconomic status * PC1 | 1.34  1.23  1.01  0.18  1.14 | 0.67, 2.71  0.51, 2.93  0.33, 3.11  0.02, 1.47  0.47, 2.75 |
|  | Relative food environment advantage  Q2  Q3  Q4  Socioeconomic status^1^  Socioeconomic status * PC2 | 0.91  0.49  0.72  0.11  1.47 | 0.45, 1.85  0.23, 1.05  0.35, 1.49  0.01, 1.25  0.57, 3.75 |
|  | Low density and high distance to accessible green  Q2  Q3  Q4  Socioeconomic status^1^  Socioeconomic status * PC3 | 1.05  1.60  0.91  0.24  0.98 | 0.52, 2.16  0.83, 3.08  0.43, 1.94  0.03, 2.41  0.47, 2.03 |
|  | Low-quality, agricultural/inaccessible green  Q2  Q3  Q4  Socioeconomic status^1^  Socioeconomic status * PC4 | 1.07  1.09  1.24  0.50  0.79 | 0.47, 2.46  0.44, 2.71  0.44, 3.48  0.05, 5.37  0.37, 1.17 |
| *Obese* |  | *OR* | *95% CI* |
|  | High food, low FEHI, high walkability  Q2  Q3  Q4  Socioeconomic status^1^  Socioeconomic status * PC1 | 1.49  1.26  1.86  0.49  0.70 | 0.65, 3.42  0.47, 3.36  0.57, 6.10  0.06, 3.93  0.26, 1.86 |
|  | Relative food environment advantage  Q2  Q3  Q4  Socioeconomic status^1^  Socioeconomic status * PC2 | 0.53  0.60  0.71  0.02  **3.47*** | 0.31, 1.59  0.25, 1.42  0.22, 1.26  0.00, 0.23  1.22, 9.90 |
|  | Low density and high distance to accessible green  Q2  Q3  Q4  Socioeconomic status^1^  Socioeconomic status * PC3 | 1.32  1.55  0.94  0.14  1.29 | 0.60, 2.91  0.74, 3.23  0.39, 2.23  0.01, 1.41  0.59, 2.79 |
|  | Low-quality, agricultural/ inaccessible green  Q2  Q3  Q4  Socioeconomic status^1^  Socioeconomic status * PC4 | 1.37  1.73  2.06  0.36  0.90 | 0.52, 3.59  0.62, 4.82  0.68, 6.25  0.06, 3.93  0.26, 1.86 |

^1^Measure of socioeconomic status at the local level, based on a combination of data regarding financial prosperity, educational level and recent employment history of the households.

***Table A4e. Interaction Effects of Socioeconomic Status and Principal Component on total DHD-index score***

| Variables | Multivariate model 2 |  |
| --- | --- | --- |
|  | β | 95% CI |
| High food, low FEHI, high walkability  Q2  Q3  Q4  Socioeconomic status^1^  Socioeconomic status * PC1 | -1.21  -2.55  -2.18  -1.62  -0.69 | -5.91, 3.49  -7.69, 2.59  -7.04, 2.68  -21.04, 17.79  -6.59, 5.22 |
| Relative food environment advantage  Q2  Q3  Q4  Socioeconomic status^1^  Socioeconomic status * PC2 | -0.33  -1.12  -3.76  -7.51  1.53 | -5.10, 4.44  -6.10, 3.85  -8.69, 1.17  -25.64, 10.62  -4.69, 7.75 |
| Low density and high distance to accessible green  Q2  Q3  Q4  Socioeconomic status^1^  Socioeconomic status * PC3 | -0.91  -0.52  -1.36  -7.97  1.85 | -5.54, 3.72  -4.89, 3.85  -5.97, 3.24  -20.33, 4.39  -2.84, 6.54 |
| Low-quality, agricultural/inaccessible green  Q2  Q3  Q4  Socioeconomic status^1^  Socioeconomic status * PC4 | -3.44  -2.06  -3.03  -8.29  2.01 | -8.43, 1.55  -6.96, 2.84  -7.68, 1.63  -22.04, 5.46  -2.82, 6.84 |

^1^Measure of socioeconomic status at the local level, based on a combination of data regarding financial prosperity, educational level and recent employment history of the households.

***Table A4f. Interaction Effects of Socioeconomic Status and Principal Component on Total Minutes of Weekly Physical Activity***

| Variables | Multivariate model 2 |  |
| --- | --- | --- |
|  | β | 95% CI |
| High food, low FEHI, high walkability  Q2  Q3  Q4  Socioeconomic status^1^  Socioeconomic status * PC1 | -0.93  -0.49  2.61  2.38  1.95 | -4.55, 2.70  -4.45, 3.46  -1.14, 6.35  -12.56, 17.32  -2.62, 6.53 |
| Relative food environment advantage  Q2  Q3  Q4  Socioeconomic status^1^  Socioeconomic status * PC2 | -1.21  -0.05  -1.48  -3.05  4.10 | -4.87, 2.45  -3.87, 3.77  -5.31, 2.35  -17.24, 11.14  -0.75, 8.94 |
| Low density and high distance to accessible green  Q2  Q3  Q4  Socioeconomic status^1^  Socioeconomic status * PC3 | -1.65  -1.30  -2.00  8.90  -0.43 | -5.23, 1.94  -4.67, 2.07  -5.56, 1.56  -0.84, 18.64  -4.11, 3.24 |
| Low-quality, agricultural/inaccessible green  Q2  Q3  Q4  Socioeconomic status^1^  Socioeconomic status * PC4 | -0.94  -1.89  2.10  9.39  -0.19 | -4.77, 2.89  -5.65, 1.87  -1.47, 5.67  -1.39, 20.18  -3.94, 3.56 |

^1^Measure of socioeconomic status at the local level, based on a combination of data regarding financial prosperity, educational level and recent employment history of the households.
